# The Effect of Polybutylcyanoacrylate Nanoparticles as a Protos Delivery Vehicle on Dental Bone Formation

**DOI:** 10.3390/ijms22094873

**Published:** 2021-05-05

**Authors:** Li-Ching Chang, Chiu-Yen Chung, Chun-Hui Chiu, Martin Hsiu-Chu Lin, Jen-Tsung Yang

**Affiliations:** 1 Department of Dentistry, Chang Gung Memorial Hospital, Chiayi 61363, Taiwan; liching@ms39.hinet.net; 2 Department of Nursing, Chang Gung University of Science and Technology, Chiayi 61363, Taiwan; 3 Department of Neurosurgery, Chang Gung Memorial Hospital, Chiayi Branch, 6, Sec. West, Chai-Pu Road, Pu-Tz City, Chia-Yi 61363, Taiwan; yen5103106@gmail.com; 4 Graduate Institute of Health-Industry Technology, Research Center for Food and Cosmetic Safety, College of Human Ecology, Chang Gung University of Science and Technology, Tao-Yuan 33303, Taiwan; chchiu@mail.cgust.edu.tw; 5 Department of Traditional Chinese Medicine, Keelung Chang Gung Memorial Hospital, Keelung 20401, Taiwan; 6 College of Medicine, Chang Gung University, Tao-Yuan 33302, Taiwan

**Keywords:** strontium, bone formation, polybutylcyanoacrylate, nanotechnique

## Abstract

Background: Dental implants are commonly used for missing teeth, for which success depends heavily on the quality of the alveolar bone. The creation of an ideal implant site is a key component in shortening the treatment time, which remains clinically challenging. Strontium ranelate (Protos) is an anti-osteoporotic agent which has previously been used to promote bone formation, however the systemic use of Protos has been linked to serious cardiovascular and venous thromboembolic events, thus local delivery strategies may be better suited for this purpose. In this study, a biodegradable, and biocompatible nanocarrier “polybutylcyanoacrylate” (PBCA) loaded with strontium was constructed and its ability to promote bone formation was assessed. Methodology: PBCA nanoparticles loaded with strontium (PBCA-Sr NPs) were synthesized using the emulsion polymerization method, and their physical properties (zeta potential, size and shape) and entrapment efficiency were characterized. Committed MSCs (osteoblasts) were derived from the differentiation of cultured rat mesenchymal stem cells (MSC), which were tested with the PBCA-Sr NPs for cytotoxicity, inflammatory response, bone formation and mineralization. Scanning electron microscopy was performed following a 7-day treatment of PBCA-Sr NPs on decellularized procaine mandibular bone blocks grafted with osteoblasts. Results: Spherical PBCA-Sr NPs of 166.7 ± 2.3 nm, zeta potential of −1.15 ± 0.28 mV with a strontium loading efficiency of 90.04 ± 3.27% were constructed. The presence of strontium was confirmed by energy-dispersive X-ray spectroscopy. Rat committed MSCs incubated in PBCA-Sr NPs for 24 hrs showed viabilities in excess of 90% for concentrations of up to 250 ug/mL, the cellular expression of osteocalcin and alkaline phosphatase were 1.4 and 1.3 times higher than the untreated control, and significantly higher than those treated with strontium alone. Bone formation was evident following osteoblast engraftment on the decellularized procaine mandibular bone block with PBCA-Sr NPs, which appeared superior to those treated with strontium alone. Conclusion: Treatment of committed MSCs with PBCA-Sr NPs showed higher expression of markers of bone formation when compared with strontium alone and which corresponded to greater degree of bone formation observed on the 3-dimensinal decellularized procaine mandibular bone block. Further quantitative analysis on the extent of new bone formation is warranted.

## 1. Introduction

Dental implant therapy is a common reconstructive technique of replacing missing tooth or teeth. The implant fixature must be placed in alveolar bone with sufficient bone quantity and quality [1,2,3]. furthermore, shortening the treatment time by hastening the development of an ideal implant site poses a great challenge in modern implant rehabilitation [4,5,6]. It can be clinically challenging to improve bone density [7], therefore new methods are required to optimize the implant site bone quality.

The bone mineral structure, Ca_10_ (PO_4_)(_6_)(OH)_2_, is approximated by the formula: (Ca,X)(_10_)(PO_4_,HPO_4_,CO_3_)(_6_)(OH,Y)_2_, where X are cations that can be substituted by cations other than calcium ions [8]. Therefore, it is possible to change the bone density by replacing calcium ions with other cations, including strontium. Strontium ranelate (Protos), which is an oral anti-osteoporotic agent, has been shown to decrease bone resorption and increase bone formation in vitro and in vivo (dual action bone agent) [9,10]. In addition, the substitution of calcium ion by strontium is possible, which could alter the calcium ion concentration in bone to affect bone cell adhesion, proliferation and morphology [11,12]. Thus, the interaction between strontium and calcium on biological properties of resident cells would be taken into consideration for bone formation. In previous studies, systemic use of Protos could promote bone formation in healthy rats and ovariectomized rats [13,14,15]. However, Protos has been taken off the shelf due to its cardiac-vascular risk [16,17,18,19].

The local use of strontium for promoting bone growth usually involves the incorporation of strontium into bone grafts [20,21,22]. Other strontium delivery methods include strontium associated with collagen sponge [23], strontium-containing collagen membrane and strontium dissolved in gel [24,25,26,27]. However, these methods are essentially matrices acting as reservoirs of drugs which are released through biolysis. Apart from the promotion of bone growth, strontium has bactericidal effects on *Staphylococcus aureus* and *Streptococcus faecalis* when used locally [28,29,30]. The bactericidal effect of strontium could improve outcome of dental implant therapy and implant-associated surgery because infection would interfere with bone formation and implant osteointegration. Additionally, the localized injection of strontium in a rat study revealed neither adverse effects on the general health of the animal, nor at the injection site [31]. However, earlier studies showed that a low oral dose of strontium promotes bone growth, but prevents bone formation at a higher dose [32,33,34]. The optimum concentration of strontium ranelate on bone cell growth is around 0.1 mM [35]. Therefore, the optimal level of strontium in local delivery is also very important for the desired effect.

Because of the properties in biodegradation, biocompatibility, low toxicity, and stability, nanocarriers are often used as drug delivery vehicles. Polybutylcyanoacrylate (PBCA) is a polymeric colloidal carrier system which is widely used in drug delivery in various settings such as central nervous system drug delivery, antiretroviral drug delivery, cancer therapy and osteointegration [36,37,38,39,40,41,42]. In addition, the nanotechnology-based delivery system can potentially overcome the pharmacokinetics to provide a slow but long-term release of drugs and easy uptake by cells [41,43,44]. In this study PBCA nanoparticles containing strontium are constructed as a local delivery strategy that differed from previous methods, we hypothesize that the new material will promote bone formation in vitro.

## 2. Results

### 2.1. Characterization of Nanoparticles

The particle sizes and zeta potential of PBCA NPs and PBCA-Sr NPs were analyzed by Zetasizer Nano ZS90 (Malvern, Worcestershire, UK), and the calculated loading efficiencies of strontium of nanoparticles are shown in Table 1. The mean diameter of PBCA NPs was measured at 126.1 ± 0.8 nm. The encapsulation of strontium ranelate into PBCA NPs increased the mean diameter to 166.7 ± 2.3 nm. The zeta potential measurements of PBCA NPs (−1.93 ± 0.24 mV) and PBCA-Sr (−1.15 ± 0.28 mV) essentially remained nearly neutral in charge. The acid emulsion polymerization method of nanoparticle synthesis produced a mono-dispersed sample with an entrapment efficiency for strontium ranelate of more than 90%.

**Figure 1:**
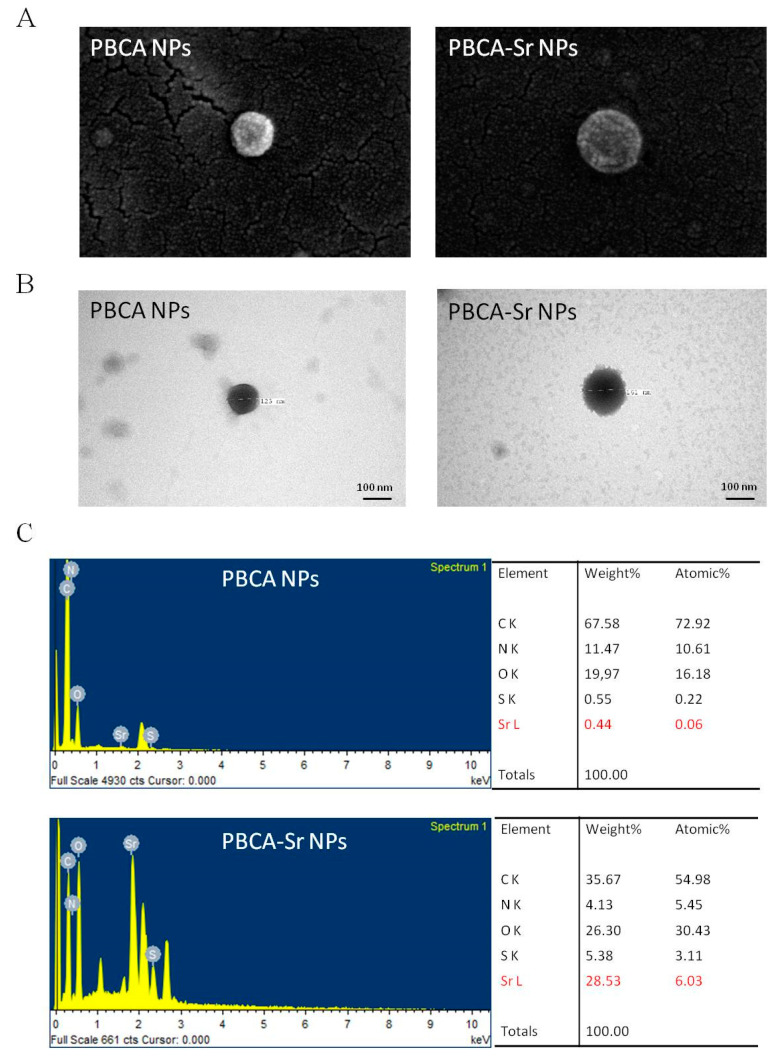
Analysis of PBCA-Sr. (**A**) FE-SEM images of PBCA NPs and PBCA-Sr NPs. Magnification: 200,000×. (**B**) TEM images of PBCA NPs and PBCA-Sr NPs. Magnification: 200,000×. (**C**) EDS analysis for PBCA NPs and PBCA-Sr NPs.

The morphology, geometry, and the elemental composition of the NPs were acquired by field emission scanning electron microscopy (FE-SEM, SU8220, Tokyo, Japan), transmission electron microscopy (TEM, H-7500, Hitachi, Tokyo, Japan), and energy-dispersive spectrometry (EDS). The results showed that both PBCA NPs and PBCA-Sr NPs had a uniform spheroid shape (Figure 1A). PBCA NPs were dark with smooth boundaries (Figure 1B); whereas PBCA-Sr NPs displayed rough and irregular boundaries. Meanwhile, PBCA NPs were smaller than PBCA-Sr NPs. The result indicated an increase in weight percentage of strontium corresponded to an increase in the size of PBCA NPs. To confirm the encapsulation of strontium into PBCA NPs, PBCA-Sr NPs were analyzed by EDC, which exhibited the presence high intensity of O, S and Sr elemental peaks, and low intensity of C and N elemental peaks when compared with PBCA NPs. This significant increase in O, S and Sr atomic percentage was taken as evidence for the existence of Sr on outer surface layer (10 nm thickness) of PBCA-Sr NPs (Figure 1C).

### 2.2. Morphology and Differentiation Potential of the Rat MSCs

**Figure 2:**
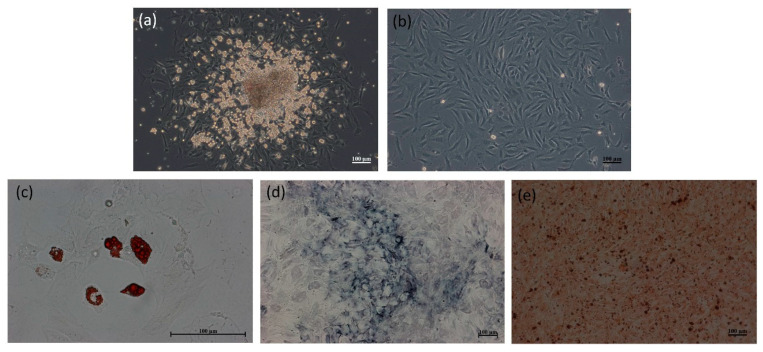
The optical images of rat bone marrow stromal cells (MSCs) at passsage-1 (**a**) and passsage-4 (**b**). Differentiation of rat MSCs into adipocytes and osteoblasts. Adipogenic MSC formed phase-bright vacuoles and were stained with oil red O (**c**). ALP staining (**d**) and Alizarin Red S staining (**e**) of osteogenic rat MSCs. Scale bar = 100 μm.

Primary rat bone marrow stromal cells (MSCs) were cultured from the marrows from femurs and tibiae of 5-week-old Sprague-Dawley (SD) rats (BioLasco, Taipei, Taiwan) to generate osteoblasts. The morphology of rat MSCs at passage-1 appeared as discrete colonies similar to ESC aggregates that exhibited a clear boundary, bright large nucleoli and scant cytoplasm in (Figure 2a). The rat MSCs colonies were detached from the cell cluster by subculture over a number of passages. Rat MSCs were morphologically homogeneous, spindle-shape and fibroblast-like at pssage-4 (Figure 2b).

To confirm the multi-lineage differentiation of rat MSCs, we assayed for adipogenesis and osteogenesis in vitro. As shown in Figure 2c, after exposure to an adipogenic stimulus for 7 days, morphologic changes in the cells as well as the formation of neutral lipid droplets in the cytoplasm that stained red with Oil Red O were noted. As shown in Figure 2d, after 7 days of culture in an osteogenic medium, morphologic changes in the cells from spindle-shape to a flattened and broadened shape occurred following the induction period. Osteogenic cells were positive for ALP staining. As shown in Figure 2e, after 3 weeks of induced osteogenic differentiation, MSC developed into committed MSCs (osteoblastic cells), as judged by their ability to mineralize the extracellular matrix which was positive for Alizarin Red S staining.

### 2.3. Assessment of Cytotoxicity

**Figure 3:**
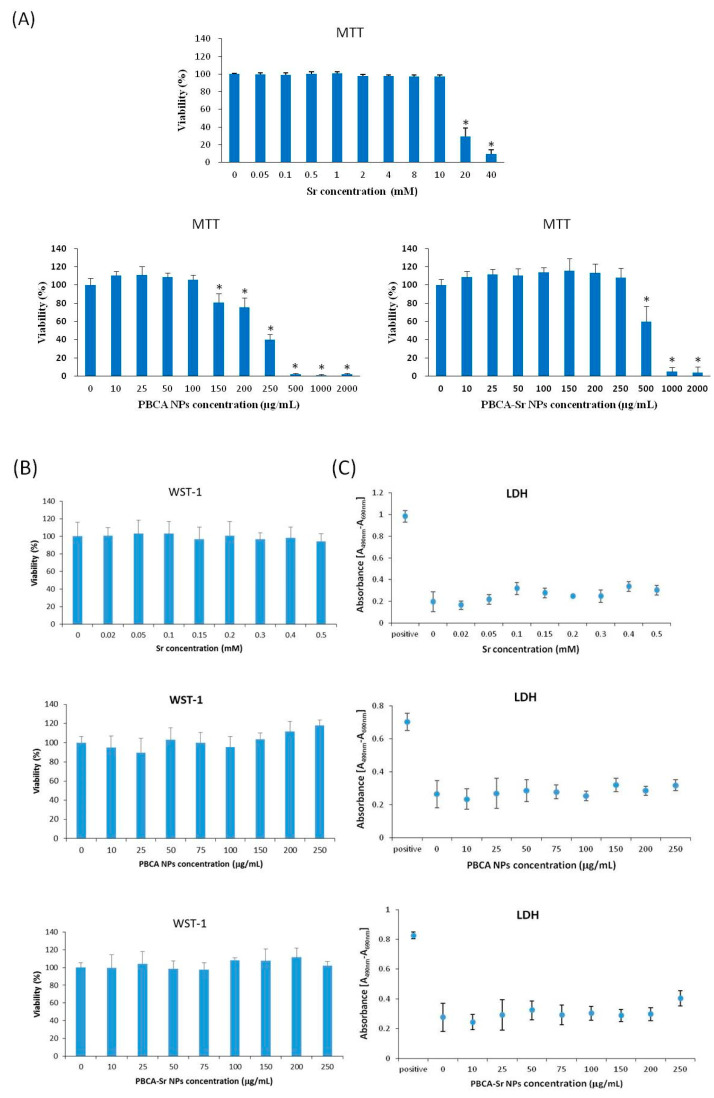

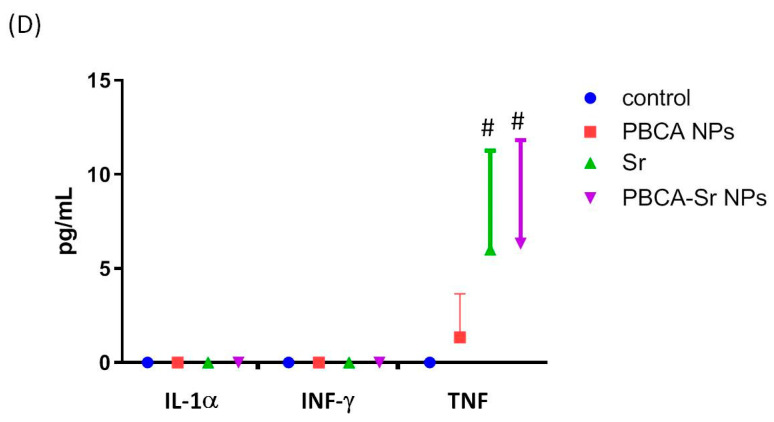
Cell viability ((**A**). MTT assay), cell proliferation ((**B**). WST-1 assay) and cytotoxicity ((**C**). LDH assay) on committed MSCs treated with strontium ranelate (Sr), and inflammatory cytokines (**D**), PBCA NPs or PBCA-Sr NPs for 24 h. *n* = 6; * *p* < 0.001, ^#^ *p* < 0.05.

Cell viability, cell proliferation and cytotoxicity of Sr, PBCA NPs and PBCA-Sr NPs to osteoblasts were estimated by the MTT assay, WST-1 and LDH assays. A concentration-dependent cell viability profile of Sr, PBCA NPs and PBCA-Sr NPs were determined by the MTT assay following 24 h of culture are shown in Figure 3A. The viability of committed MSCs maintained over 90% with Sr concentrations of 0.05 to 10 mM, but Sr concentrations of greater than 10 mM led to significant reductions in viability. PBCA NP concentration in excess of 100 μg/mL and PBCA-Sr NPs concentration of more than 250 μg/mL were found to be cytotoxic to the osteoblasts. The cell proliferation assay was carried out using the WST-1 assay on committed MSCs cultured with different concentrations of NPs for 24 h. A concentration-dependent cell proliferation profile of Sr, PBCA NPs and PBCA-Sr NPs are shown for Figure 3B. The result showed the viability is more than 90% at Sr concentrations of up to 0.05 mM, PBCA NP concentrations of up to 250 μg/mL, and PBCA-Sr NP concentrations of up to 250 μg/mL. Toxicity testing was carried out by the LDH assay following 24 h of culture are shown in Figure 3C. The positive control is the amount of LDH released from the lysed committed MSCs which was taken as “100% toxicity”. The negative control is the amount of LDH released from untreated committed MSCs which was denoted as “0% toxicity”. There was no significant difference between any of the concentrations of Sr, PBCA NPs or PBCA-Sr NPs compared with negative control. The result showed no significant toxicity at Sr concentrations of up to 0.05 mM, PBCA NP concentrations of up to 250 μg/mL, and PBCA-Sr NP concentrations of up to 250 μg/mL. On the other hand, the inflammatory cytokines IL-1α, IFN-γ and TNF were assessed by the CBA kit. Except for TNF, both IL-1α, IFN-γ were not detected when the Sr concentration of 0.2 mM, similar changes were detected on PBCA-Sr NP with a concentration of 100 μg/mL.

### 2.4. The effect of Osteogenesis and Mineralization of PBCA-Sr NPs on Committed MSCs

#### 2.4.1. 2D Culture

**Figure 4:**
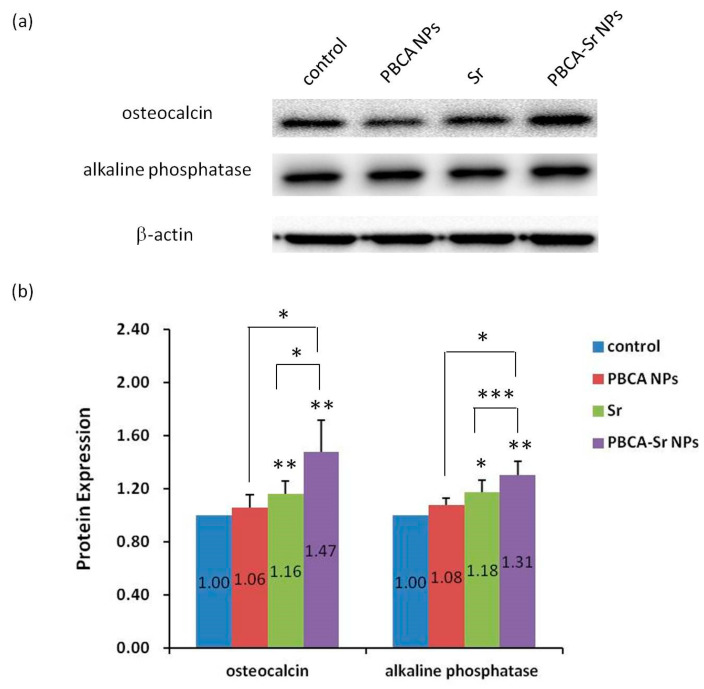
(**a**) Western blot analysis of osteocalcin and alkaline phosphatase at day-7 after treated with PBCA NPs (50 μg/mL), strontium ranelate (0.1 mM) or PBCA-Sr NPs (50 μg/mL containing 0.1 mM Sr). β-actin was used as a loading control. (**b**) Quantitative analysis was presented. The expression level of osteocalcin and alkaline phosphatase protein were normalized to control of β-actin. * *p* < 0.05, ** *p* < 0.01 and *** *p* < 0.001.

In order to demonstrate the effect of strontium-loaded nanoparticles on osteogenesis, we examined the expression of osteocalcin and alkaline phosphatase on committed MSCs at day-7 after treatment with PBCA NPs (50 μg/mL), Sr (0.1 mM) or PBCA-Sr NPs (50 μg/mL containing 0.1 mM Sr) by western blotting analysis. As shown in Figure 4, the protein expression levels of osteocalcin and alkaline phosphatase increased significantly in the cells treated with PBCA-Sr NPs.

**Figure 5:**
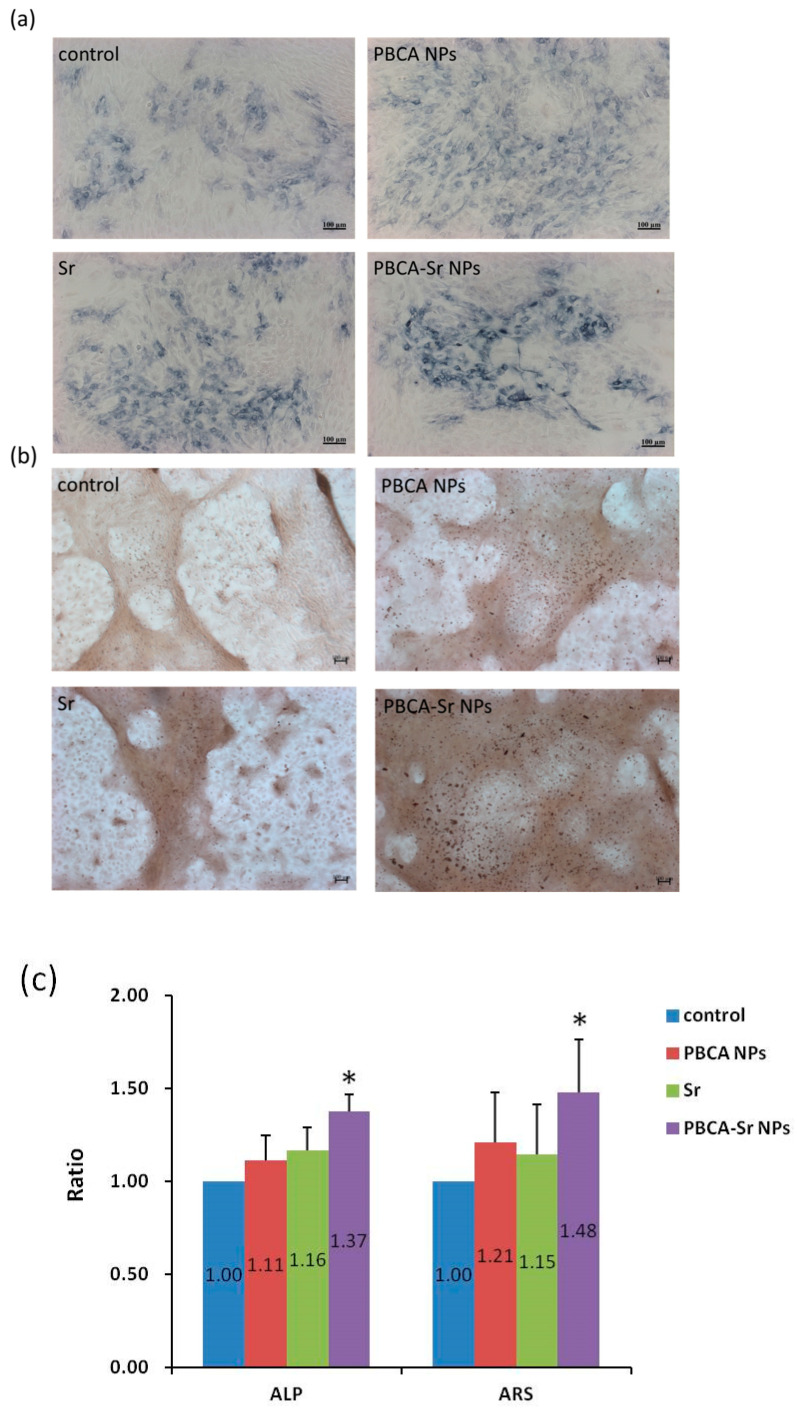
Influence of Sr-NPs on committed MSCs. Committed MSCs was treated with strontium ranelate (0.1 mM), PBCA NPs (50 μg/mL) or PBCA-Sr NPs (50 μg/mL containing 0.1 mM Sr). (**a**) Alkaline phosphatase staining following exposure medium for 7 days. (**b**) Alizarin Red S staining was performed at 21 days after treatment. Scale bar = 100 μm. (**c**) Quantification of ALP and ARS as a ratio to control. * *p* < 0.05.

Moreover, committed MSCs (1 × 10^4^ cells/cm^2^) were treated with PBCA NPs (50 μg/mL), Sr (0.1 mM) or PBCA-Sr NPs (50 μg/mL containing 0.1 mM Sr) and incubated for 7 and 21 days. Alkaline phosphatase (ALP) staining and Alizarin Red S (ARS) staining were then performed separately. ALP staining demonstrated that the intensity of ALP activity appeared higher for PBCA-Sr NPs after 7 days (Figure 5a), this was also true for ARS staining (Figure 5b). The quantified staining intensity showed significant increases in ALP and ARS stain intensity for PBCA-Sr-NPs over Sr, PBCA-NPs and control (Figure 5c).

#### 2.4.2. 3D Culture

**Figure 6:**
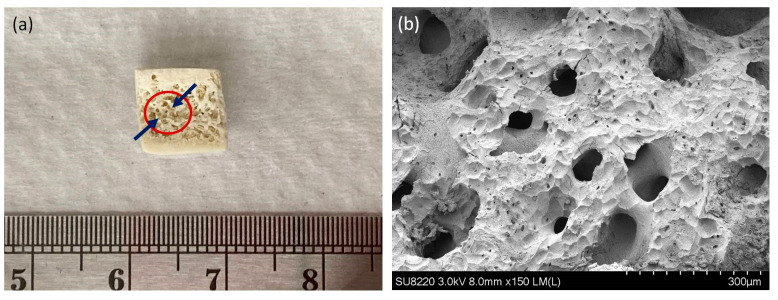
The morphology of procaine mandible bone block (**a**). FE-SEM images of procaine mandible bone block (**b**). Magnification: 150×.

A 3-D bone model produced by the decellularization of procaine mandible bone block was used to prove that the Sr-NP promote bone formation. Figure 6 shows the SEM images of a decellularized procaine mandible bone block. As indicated in this figure, the pore structure of bone block with different size is clearly evident. The morphology indicated that the typical trabecular structure of cancellous bone is well preserved even after decellularization. The committed MSCs engrafted bone blocks treated with PBCA-Sr NPs appeared to have better bone matrix deposition as indicated by the roughened surface texture and the coverage of pores less than 30 microns.

**Figure 7:**
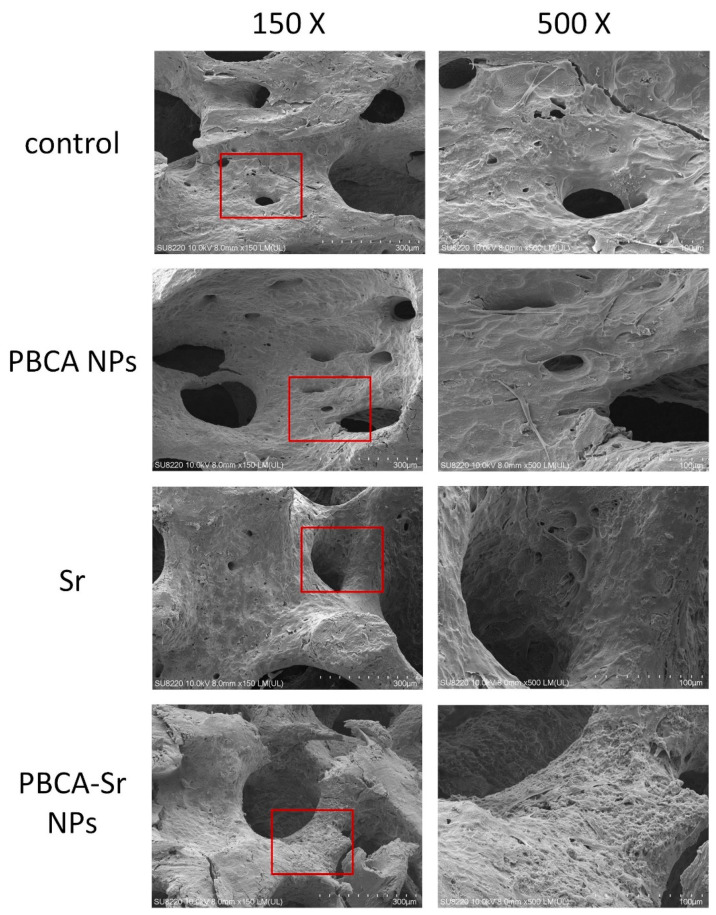
FE-SEM images of committed MSCs in procaine mandible bone block. Cells were treated with PBCA NPs (50 μg/mL), strontium ranelate (0.1 mM) or PBCA-Sr NPs (50 μg/mL containing 0.1 mM Sr) for 7 days. Areas highlighted in the red square are displayed under higher magnification on the right side. Magnification: 150× and 500×.

In Figure 7, the SEM images shows that the committed MSCs attached on the bone block have similar morphology at day-7 after treated with PBCA NPs (50 μg/mL), Sr (0.1 mM) or PBCA-Sr NPs (50 μg/mL containing 0.1 mM Sr).

## 3. Discussion

Strontium (Sr) is a promising trace element that can trigger new bone formation by inducing osteoblasts and preventing osteoclast activity. Strontium ranelate is a kind of strontium-containing anti-osteoporotic drug, which can significantly reduce the probability of fractures in patients with osteoporosis [45]. However, concerns over serious cardiovascular and venous thromboembolic events associated with the systemic administration of Protos has led to the exploration of local delivery strategies [46,47].

In this study, we have successfully synthesized and characterized a novel nanocarrier “polybutylcyanoacrylate nanoparticle” loaded with strontium ranelate as a local drug delivery system by the emulsion polymerization method [48]. The PBCA-Sr NPs were spherical with diameters of 166.7 ± 2.3 nm, zeta potential of −1.15 ± 0.28 mV, and strontium ranelate loading efficiency of 90.04 ± 3.27%. The incorporation of strontium ranelate in the PBCA nanoparticles leads to an increase in particles size, but does not affect the zeta potential.

As a series of in vitro and in vivo studies have shown, strontium ranelate has been demonstrated to modulate bone formation and bone resorption [49], accordingly, it has been show that different strontium concentrations have different effects on bone formation in vitro; for bone-forming cells, the most effective concentration is between 0.001 and 1 mM, and for the reduction of bone resorbing cells, the most effective concentration is between 0.01 and 1 mM [50]. In our study, the concentration of strontium incorporated into PBCA nanoparticle is within this range. Strontium has been proven to be an anti-inflammatory drug [51]. In this study, when the Sr concentration was 0.2 mM, the PBCA NP concentration was 100 μg/mL, and the PBCA-Sr NP concentration was 100 μg/mL, the inflammatory cytokines IL-1α, IFN-γ were undetected, with only low levels of TNF induced, which is consistent with those reported in literature on the anti-inflammatory effect of Sr [52,53]. Strontium and calcium (Ca) in bones have similar cellular and physiochemical properties. Although the exact mechanism of Sr in bone is not clear, it has been proposed that Sr interacts with cellular targets by activating calcium-sensing receptor (CaSR) similar to Ca^2+^, thereby acting as a signaling transduction pathway related to Ca-driven regulation of bone metabolism [54]. The phosphatase activity (ALP) is one of the most commonly used markers for osteoblast differentiation, and the enzyme activity is considered a necessary prerequisite for bone mineralization. The role of osteocalcin is bone mineralization and calcium ion homeostasis [55]. Strontium induces the higher expression of osteoblastic genes such as alkaline phosphatase (ALP), osteocalcin (OCN) and bone sialoprotein, which is accompanied by an increase in bone nodules and a decrease in the number of mature osteoclasts in vitro [51]. Here, we demonstrated that culturing osteoblasts in vitro with strontium-containing nanomaterials can meaningfully increase the expression of osteogenic proteins such as ALP and OCN, and increase the formation of calcium deposits.

In order to understand the effect of Sr-NP on the growth of osteoblast in the 3-D bone model, procaine mandible bone block decellularization was use to guide bone regeneration. Similar to the expression of osteogenic proteins, the results showed greater degree of bone matrix deposition on the raw surface of the trabecular bone following treatment with PBCA-Sr NP as indicated by the roughened ultrasturactural surface texture of the bone blocks and the coverage of pores less than 30 microns on the trabecular bone.

## 4. Materials and Methods

### 4.1. Synthesis of PBCA-Sr NPs

PBCA NP was synthesized by the emulsion polymerization method with minor modification [48]. 1% (*v*/*v*) butylcyanoacrylate (BCA, Sicomet, Sichel Werk, Hanover, Germany) monomers were added drop by drop into 0.1 N HCl acidic polymerization medium containing 1% (*w/v*) dextran 70,000 (Sigma, St. Louis, MO, USA) and 0.5135 g (100 mM) strontium ranelate (Protos^®^, Servier, France) at pH 2.0 (pH adjusted with 12 N HCl) under 400 rpm and 25 °C for 3 h. 0.1 N NaOH was mixed with NP suspension to terminate polymerization, and then the suspension was centrifuged at 5250× *g* for 10 min. The larger polymer aggregates were separated from the nanoparticles suspension by filtration through 0.22 μm filtration units. The drug-free NPs were prepared using the same method.

### 4.2. Characterization

The concentration of PBCA-Sr NPs in DPBS buffer at pH 7.4 was 2 mg/mL in characterization analysis. The particle size and zeta potential of PBCA-Sr NPs were determined by Zetasizer Nano ZS90 (Malvern, Worcestershire, UK) with photo correlation spectroscopy. The geometry, surface morphology and the elemental composition of NPs were obtained by a transmission electron microscope (TEM, H-7500, Hitachi, Tokyo, Japan), field emission scanning electron microscope (FE-SEM, SU8220, Tokyo, Japan) and energy-dispersive spectrometry (EDS).

### 4.3. Evaluation of Entrapment Efficiency

The free drug in the supernatant was measured using inductively coupled plasma mass spectroscopy (NexION 350X ICP-MS, PerkinElmer, Waltham, MA, USA). The entrapment efficiency of Sr, *E*_e_, is defined as *E*_e_ (%) = (Total weight of Sr−weight of Sr in supernatant)/ (Total weight of Sr) × 100%.

### 4.4. Isolation of Rat Bone Marrow Stromal Cells

The primary bone marrow stromal cells (MSCs) were from the long bone marrow of 5-week-old Sprague-Dawley (SD) rats (BioLasco, Taipei, Taiwan). The femurs and tibiae were aseptically removed from the animals and dissected clean of attached muscles. Bone marrow were flushed with 10 mL of cold PBS with 1% FBS and cells were washed 2–3 times with 1X PBS. Then, cells were cultured with α-MEM containing 10% define FBS, antibiotics (100 U/mL penicillin G and 100 mg/mL streptomycin sulfate, Invitrogen, California). Flasks were incubated in a humidified atmosphere with 5% CO_2_ at 37 °C. After 8 days, nonadherent cells were removed and remaining adherent cells were detached by trypsinization. When the culture is 80–90% confluence, the cells were passaged at the ratio of 1:4 in 75-cm^2^ tissue culture flasks. Cells from the 3th to the 4th (18–24 days) passage were used for this study.

### 4.5. MSC Differentiation Potential

To induce adipogenesis, fourth-passage cells were seeded in 6-well plates at 2 × 10^4^ cells/cm^2^ and cultured in growth medium overnight. Cultures were then treated with Complete Adipogenesis Differentiation Medium (StemPro^®^ Adipogenesis Differentiation Kit, Gibco, Carlsbad, CA, USA) for 7 days. Refeed cultures twice weekly and adipogenesis was assessed at day 7. Cells were fixed with 4% paraformaldehyde and stained with Oil Red O (Sigma) to evaluate adipogenesis via the accumulation of neutral lipids.

To induce osteogenic differentiation, fourth-passage cells were seeded in 6-well plates at 2 × 10^3^ cells/cm^2^ and cultured in growth medium overnight. Cultures were then treated with osteogenic medium consists of DMEM containing 10% FBS, antibiotics (100 U/mL penicillin G and 100 mg/mL streptomycin sulfate), 100 nM Dexamethasone (Sigma, Saint Louis), 10 mM β-glycerophosphate (Sigma) and 50 μM L-ascorbate-2-phosphate (Sigma) for 1 week and 3 weeks. Medium was replaced twice weekly. Osteogenic differentiation was revealed after 1 week by ALP accumulation, as detected by microscopy after staining with BCIP/NBT Liquid Substrate System (Sigma-Aldrich). In the other hand, On week 3 of osteogenic differentiation, the cells were stained using 2% alizarin red (Sigma) solution (pH 4.2) to stain the calcium deposits.

### 4.6. Differentation of Rat Committed MSCs and Cell Culture

Rat MSCs were seeded with a density of 1 × 10^4^ cells/cm^2^ and incubated overnight. Then, the medium was replaced with osteogenic medium consists of DMEM containing 10% FBS, antibiotics (100 U/mL penicillin G and 100 mg/mL streptomycin sulfate), 100 nM Dexamethason (Sigma, Saint Louis), 10 mM β-glycerophosphate (Sigma) and 50 μM L-ascorbate-2-phosphate (Sigma) in a humidified CO_2_ incubator at 37 °C. When the culture is 80–90% confluence, the cells were passaged at the ratio of 1:4 in 75-cm^2^ tissue culture flasks. Cells from the 3th to the 4th passage were used for this study.

### 4.7. Evaluate the Effect of PBCA-Sr NPs on Osteoblasts in Vitro

#### 4.7.1. Cell Viability Test

##### MTT Assay

The cytotoxicity of PBCA-Sr NPs to committed MSCs was estimated by 3-(4,5-dimethylthiazol-2-yl)-2,5- diphenyltetrazolium bromide (MTT) assay (Sigma). Cells were seeded into a 96-well plate at a density is 10,000 cells/well and incubated overnight. Then the cells were treated with different concentrations of NPs in 100 μL growth medium and are incubated for 24 h. Then, the cells were incubation with 100 μL of MTT solution (1 mg/mL) for 2 h at 37 °C in a humidified atmosphere of 5% CO_2_ incubator. MTT solution was removed and replace with dimethylsuphoxide (DMSO, Sigma-Aldrich) to solubilize the blue formazan crystals. The optical absorbance was measured by an EnSpire Multimode Plate Reader (Perkin Elmer Inc., Waltham, MA, USA) at wavelength of 570 nm. Percentage of cell viability was defined as the relative absorbance of PBCA-Sr NPs -treated cells versus the control cells. The percentage of the absorbance of control cells was taken as 100% viability.

##### WST-1 Assay

WST-1 assay was used to investigate the cell proliferation. Cells were seeded on 96-well plate at a density is 10,000 cells/well and incubated overnight. The cell viability was estimated after cells exposure to different concentration of NPs for 24 h by WST-1 assay (cell proliferation kit, Roche). The absorption is measured by a spectrophotometer (ELISA reader) at a wavelength of 450 nm.

##### LDH Assay

Cells were seeded on 96-well plate at a density is 10,000 cells/well and incubated overnight. Then, cells were added with different concentrations of NPs. After 24 h, the cytotoxicity was estimated by LDH assay (cytotoxicity detection kit, Roche). The absorption is measured by a spectrophotometer (ELISA reader) at a wavelength of 490 nm.

##### Determination of Inflammation

The committed MSCs are seeded into a 96-well plate at a density is 1 × 10^4^ cells/cm^2^ and incubated overnight. The cell medium was collected after treating with different concentrations of NPs for analyzing IL-1α, IFN-γ and TNF-α by BD cytometric bead array (CBA) kit (BD Biosciences, NJ, USA). The kit was performed according to the manufacturer’s instructions and analyzed by flow cytometry.

#### 4.7.2. ALP Staining

The committed MSCs were treated with different concentrations of PBCA-Sr NPs for 7 days. Cells were washed with 1× PBS and fixed with 4% formaldehyde for 20 min at room temperature. Then, BCIP/NBT Liquid Substrate System (Sigma-Aldrich) was added for ALP staining.

#### 4.7.3. Analysis of Calcium Deposition

The committed MSCs were treated with different concentrations of PBCA-Sr NPs for 21 days. Cells were washed with 1× PBS and fixed with 4% formaldehyde for 20 min at room temperature. Then, 2% alizarin red (pH 4.2) was added for calcium staining.

#### 4.7.4. Western Blot Analysis

The committed MSCs were cultured with different concentrations of PBCA-Sr NPs for 7 days. The cells were lysed using M-PER Mammalian Protein Extraction Reagent (Thermo Fisher Scientific, Waltham, MA, USA) and centrifuged at 12,000 rpm at 4 °C for 15 min. Protein concentration was measured by Bio-Rad protein assay reagent. Protein separation was performed by using SDS polycarylamide gel electrophoresis (SDS-PAGE) and transferred onto PVDF membrane. Blocking of non-specific binding was achieved by placing the PVDF membrane in a 1% BSA blocking solution and stained with antibodies specific for osteocalcin (OCN, 1:1000, Santa Cruz Biotechnology), alkaline phosphatase (1:1000, Abcam, Cambridge, UK) or β-actin (1:10000, Santa Cruz), gently shaken in room temperature for 1 h, then washed 3 times for 15 min with washing buffer (10 mM Tris-base, pH 7.5, 100 mM NaCl 0.1% Tween 20). Secondary antibody diluted in peroxidase-containing blocking buffer is added, incubated for 1 h, then, washed 3 times for 15 min with washing buffer. The PVDF membrane was analyzed by chemiluminescent-base detection system for detection of protein expression.

### 4.8. Generation of Procaine Mandibular Bone Block by Decellularization

The procaine mandibular bone samples were harvested from maker (Chia-Yi, Taiwan). The procaine mandibular bone has been cut into a cuboid of 10 mm × 10 mm × 10 mm and stored at −80 °C until use. The bone blocks were washed with 1× PBS+ 1× PSG (antibiotics, 100 U/mL penicillin G and 100 mg/mL streptomycin sulfate). Then, bone blocks were immersed in distilled water (ddH_2_O) and performed 2 cycle of thermal shock. The solution was changed at every cycle. The step of thermal shock was heated at 121 °C for 20 min, followed by freezing in liquid nitrogen overnight. To remove cellular debris, bone blocks were washed with 1% Triton X-100 and then 0.1% Triton X-100. Bone blocks were washed with ddH_2_O to remove residual Triton X-100. Bone blocks were dehydrated in 50, 75, 95, and 100% ethanol. All these steps were performed under continuous shaking at room temperature with the rotator shaker. Bone blocks were transferred to cell culture dishes and allowed to dry at RT under a sterile laminar flow hood.

### 4.9. The Morphology of Osteoblasts Attached on Bone Block

After sterilization, pig bone blocks were transferred to 6-well culture plates. The committed MSCs (1 × 10^4^ cells/cm^2^) are seeded into bone block and incubated overnight. The cells were cultured with different concentrations of PBCA-Sr NPs for 7 days. Sample were rinsed in PBS and then fixed with 3% glutaraldehyde and 2% paraformaldehyde in 0.1 M cacodylate buffer (pH 7.4) at 4 °C for 2 h. The fixative was removed by washing with 0.1 M cacodylate buffer and post-fixed in 1% osmium tetroxide in 0.1 M cacodylate buffer at 4 °C or 1 h. Then the cells were dehydrated in a graded ethanol series (30, 50, 70, 95, and 100%). The specimens were coated with platinum and the morphology of osteoblasts attached on pig bone block is characterized by FE-SEM (SU8220).

### 4.10. Statistics

Results were presented as mean ± standard deviation. The data were analyzed with a one-way analysis of variance (ANOVA), and the comparisons between pairs were performed with Tukey HSD test. A *p* value of 0.05 or less indicated a significant statistical difference.

## 5. Conclusions

The nanoparticle-based delivery of Sr using PBCA NPs can aid bone formation by increasing osteocalcin and alkaline phosphatase protein expression and promoting calcium deposition in committed MSCs, which are superior to Sr alone. PBCA-Sr-NPs could potentially be a biocompatible delivery system used locally for optimization of bone quality in dental implantation.

## Figures and Tables

**Table 1:** The average diameter (Dav), zeta potential, polydispersity (PDI), and entrapment efficiency of strontium of nanoparticles.

Sample	*D*av (nm)	Zeta Potential (mV)	PDI	*EE* _Sr_ (%)
PBCA NPs	126.1 ± 0.8	−1.93 ± 0.24	0.044 ± 0.009	-
PBCA-Sr NPs	166.7 ± 2.3	−1.15 ± 0.28	0.241 ± 0.019	90.04 ± 3.27

*n* = 3.
